# Dysregulation of pulmonary endothelial protein C receptor and thrombomodulin in severe falciparum malaria-associated ARDS relevant to hemozoin

**DOI:** 10.1371/journal.pone.0181674

**Published:** 2017-07-21

**Authors:** Sitang Maknitikul, Natthanej Luplertlop, Georges E. R. Grau, Sumate Ampawong

**Affiliations:** 1 Department of Tropical Pathology, Faculty of Tropical Medicine, Mahidol University, Ratchathewi, Bangkok, Thailand; 2 Department of Microbiology and Immunology, Faculty of Tropical Medicine, Mahidol University, Ratchathewi, Bangkok, Thailand; 3 Vascular Immunology, Department of Pathology, Sydney Medical School, The University of Sydney, Camperdown, NSW, Australia; Institute of Tropical Medicine (NEKKEN), Nagasaki University, JAPAN

## Abstract

To investigate the role of the protein C system, endothelial protein C receptor (EPCR) and thrombomodulin (TM) in the pathogenesis of malaria-associated acute respiratory distress syndrome (ARDS) in relation to hemozoin and proinflammatory cytokines-induced type II pneumocyte injury and -aggravated pulmonary resolution. A total of 29 left-over lung specimens that were obtained from patients who died from severe falciparum malaria were examined. Histopathological, immunohistochemical and electron microscopic analyses revealed that ARDS coexisted with pulmonary edema and systemic bleeding; the severity was dependent on the level of hemozoin deposition in the lung and internal alveolar hemorrhaging. The loss of EPCR and TM was primarily identified in ARDS patients and was related to the level of hemozoin, parasitized red blood cell (PRBC) and white blood cell accumulation in the lung. Moreover, an *in vitro* analysis demonstrated that interleukin-13 and -31 and hemozoin induced pneumocytic cell injury and apoptosis, as assessed by EB/AO staining, electron microscopy and the up-regulation of *CARD*-9 mRNA (caspase recruitment domain-9 messenger-ribonucleic acid). The dysregulation of EPCR and TM in the lung, especially in those with increased levels of hemozoin, may play an important role in the pathogenesis of malaria-associated ARDS through an apoptotic pathway.

## Introduction

Malaria infection caused by *Plasmodium falciparum* is the most deadly form and it predominates in tropical countries [1]. Pulmonary complications associated with falciparum malaria are estimated to occur in 9%–23% of cases, range from mild respiratory symptoms to pulmonary edema (PE) with or without ARDS; moreover, it usually presents in conjunction with cerebral malaria (CM), acute renal failure and high parasitemia [2–5]. In the lungs of CM patients, 51% of the blood vessels exhibited PRBC sequestration in septal capillaries and small blood vessels, which was significantly higher than in the lungs of non-CM patients [6]. Several mechanisms are considered to play an important role in acute lung injury and ARDS; however, one classical phenomenon involved is the disturbance of the pulmonary microcirculation by an occlusion with PRBCs. Such alterations result in a cascade of events, including cytokine overproduction, mononuclear cell and neutrophil activation through the initiation of ischemic hypoxia and blood gas barrier leakage, which are thought to be key events [7–9].

It has been well established that coagulopathy is closely related to the pathogenesis of malaria-associated acute lung injury [10–12]. The protein C system is an important natural anticoagulant mechanism mediated by activated protein C and its receptor, the EPCR, which regulates the activity of factors VIIIa and Va. In addition, it modulates endothelial dysfunction by blocking cytokine signalling, acts as an adhesion site of *P*. *falciparum* erythrocyte membrane protein-1 (PfEMP-1), controls vascular permeability, vascular protective signals and prevents the induction of apoptosis [13]. Recently, in children who have died of CM, the endothelial sites of adherent PRBCs have been demonstrated to colocalize with the loss of EPCR and thrombomodulin [14]. These findings indicate a disruption of the protective endothelial properties in the brain, linking coagulation and inflammation with PRBC sequestration. In addition, EPCR acts as the endothelial receptor for PfEMP-1[15, 16] and provides a parasitic binding site [16, 17]. Moreover, in Thai patients, it has been reported that there is an association between the EPCR-rs867186-G allele and protection from severe malaria [18]. However, whether the loss of the protein C system is related to localised coagulopathy in the lung and leads to ARDS in association with PE in the same manner presented as in CM [14, 16, 17, 19], remains unknown.

It has been well established that the central role in ARDS pathogenesis focuses on endothelial cells. However, epithelial injury is also played an important role in term of both injury and repair mechanisms in which affected on type I and II pneumocyte, respectively. The loss of type II pneumocyte and its integrity worsen the alveolar fluid transport disturbance and the reduction of surfactant production, therefore whole alveoli collapse or completely flood are consequently observed [20–22]. The scare observation of type II pneumocyte in malaria-associated ARDS patients is still unclear. Interestingly, based on the histopathological and electron microscopic results, a positive correlation between ARDS severity and the deposition of lung hemozoin was found in our cases. Moreover, a number of pro-inflammatory cytokines have been claimed to be involved in the pathogenesis of severe falciparum malaria (SM). Level of IL-31 and IL-33 were strongly elevated in SM patients, in contrast to IL-27, which was decreased in SM patients and increased in healthy subjects [23, 24].

The present study aimed to demonstrate that in cooperation with the dysregulation of protein C system, whether the absence of type II pneumocyte relates to synthetic (s)-hemozoin or cytokines- induced apoptosis and leads to ARDS in association with PE. The study mainly focused on the clinical presentation and pulmonary histopathological changes related to ARDS with or without PE. Histopathological, immunohistochemical and electron microscopy studies were conducted. In addition, an *in vitro* model was used to assess the effect of hemozoin and interleukin (IL)-13, -27 and -31 on the lung epithelial cell line, A549. In particular, we sought to evaluate the effect of these cytokines and *P*. *falciparum* (*Pf*)-hemozoin on the integrity of the lung epithelium with an emphasis on apoptosis. Apoptotic pneumocytes were morphologically examined via electron microscopy and dual EB/AO staining. Real-time RT-PCR (reverse transcriptase-polymerase chain reaction) was used to determine the level of apoptosis regulator gene expression and caspase recruitment domain containing protein (*CARD*-9), as additional confirmation. The results of this study provided a new insight into the understanding of malaria-associated ARDS, with particular attention to underlying coagulative disorders and the effect of cytokines and hemozoin on cellular apoptosis.

## Materials and methods

### 1. Ethical statement

All the excess lung specimens and anonymous clinical data presented in this study were permitted and approved by the Hospital for Tropical Diseases and the Ethics Committee of the Faculty of Tropical Medicine, Mahidol University, Thailand (approval number MUTM2014-049-1 and -2).

### 2. Specimens and clinical data

The total of 29 lung specimens were obtained from patients who died from severe falciparum malaria at the Hospital for Tropical Diseases, Thailand from 1969 to 2004. All patients were cautiously clinically examined and diagnosed upon admission. Asexual *P*. *falciparum* blood stages were examined in the peripheral blood by blood smear. The WHO criteria for severe malaria were adopted as the presence of one or more of the following complications: cerebral malaria; jaundice; PE; renal failure; hypoglycemia; hyper-parasitemia and shock [25]. Concerning the determination of ARDS, the presence of hyaline membrane formation from the lungs was mainly used as a histopathological criterion to separate all patients who presented with or without ARDS. The pulmonary signs were additionally traced back and evaluated, particularly the clinical onset (acute; within a week or chronic), progressive respiratory symptoms (e.g. pneumonia), increased respiratory rate and intercostal indrawing with or without consolidation on the chest x-ray. Based on the lung histological examination, the patients who had cardiogenic pulmonary edema (as presented by heart failure cell or siderophage in the lung) and purulent pneumonia (as presented by oedematous fluid and intense inflammatory cells, particularly neutrophil, deposition in the alveolar sac) were excluded from the study. Together with the clinical data, the patients who had septicemia, diffuse pulmonary infection, gastric aspiration, burns, near-drowning or head injuries were also excluded from the study.

### 3. Histopathology

All lungs were preserved in 10% neutral buffered formalin. The fixed specimens were dehydrated, infiltrated, embedded and sectioned into 4–5 μm thickness. All lung sections were stained with haematoxylin and eosin and Masson’s trichrome to examine histopathological changes and the deposition of intravascular fibrin, respectively. The presentation of alveolar expansion with increasing pneumocytic cells, septal congestion with blood components (e.g. red blood cells [RBCs]; white blood cells [WBCs]; PRBCs), malarial pigment (both in PRBCs and mononuclear cells characterized by golden-brown birefringence pigment), alveolar haemorrhage, PRBC sequestration, WBC accumulation and hyaline membrane formation were scored according to four grades: 0 = absent; +1 = focal (less than 25% of the section); +2 = present (between 25% and 50% of the section) and +3 = severe (more than 50% of the section). In addition to the clinical evaluation, ARDS was confirmed by the presence of hyaline membrane formation and widespread inflammation. In addition, PE was also histologically described by the presence eosinophilic fluid in more than 25% of the alveolar spaces. Five normal lung tissues from trauma cases with no gross pathological changes at the time of the autopsy or on subsequent histological examination were included in the present study. The histopathological examination was fully blind-graded by two observers and was additionally scored by a third investigator should any disagreements arise.

### 4. Immunohistochemistry

In the present study, polyclonal rabbit anti- EPCR (SantaCruz, USA, sc-28978), anti-thrombomodulin (TM) (SantaCruz, USA, sc-9162), anti-intercellular adhesion molecule (ICAM)-1(SantaCruz, USA, sc-7891) and tumor necrotic factor (TNF)-α (Immunotool, Germany, 21453011) antibodies were used for immunohistochemical staining. Lung antigenicity was retrieved by heat applied in citrate buffer (pH 6.0), using a microwave oven. Peroxidase activity and non-specific binding were blocked within EnVision FLEX/HRP blocking buffer (DAKO, Denmark, K8002) for 10 min. The sections were incubated with a primary antibody for 1 h, washed and labelled with HRP anti-mouse/rabbit (DAKO, Denmark, K8002) for 20 min. Next, the sections were visualized with diaminobenzidine (DAB) (DAKO, Denmark, K8002), counterstained with hematoxylin and mounted with DPX (Merck, Germany).

### 5. H-score measurement

To determine the intravascular fibrin and the expression of EPCR, TM, ICAM-1 and TNF-α, the H-score was implemented. The H-score (range: 0 to 300) was calculated by the multiplication of the percentage area of expression (0%–100%) and the intensity score (4 grades: 0 = no staining; 1 = mild staining; 2 = moderate staining and 3 = strong staining). The area of expression was measured by an imaging analysis program (ImageJ^®^ Version 1.36; NIH, USA). Briefly, 10 randomly high power field images of the lungs were acquired using a digital camera (DP20, Olympus^®^, Japan) on a light microscope (BX51, Olympus^®^, Japan) and colours that were not of interest were removed via the replace mode. The adapted images were converted to greyscale and then the area of expression was located by adjusting the threshold. The percentage area of expression was denoted by the positive pixels on the labelled areas. For intravascular fibrin, the fibrin-positive area as characterized by yellowish material inside the vessel was measured, whereas the expression of EPCR, TM, ICAM-1 and TNF-α were measured by the septal area and image.

### 6. A549 cells with cytokines or s-hemozoin

An *in vitro* model of lung epithelial cells using a human lung adenocarcinoma cell line, A549 (ATCC-CCL-185), with cytokine or s-hemozoin treatment was conducted. Recombinant human (rh) IL-13, -27 and -31 (Immunotools^®^, Germany) were used to determine the protective or deleterious effect on pneumocytes due to these cytokines in relation to severe malaria. In addition, to demonstrate the pro-oxidative effect of hemozoin on pneumocytic cells, s-hemozoin (39404-00-7, InvivoGen, USA) was used. This synthetic crystal is commercially claimed that both size and physiochemical properties are similar to *Plasmodium falciparum* (Pf) hemozoin [26]. In addition, it was synthesized by acidic method and described as a devoid of protein and DNA contamination. A549 were maintained in a complete cell culture medium composed of RPMI-1640 supplemented with 10% inactivated foetal bovine serum and 1% Penicillin/Streptomycin (100 U/100 mg/mL) at 37°C in a 5% CO_2_ atmosphere. The 10^4^ cells/mL of A549 cells were seeded on a cover slip or transwell^®^ polyester membrane in 12-well plates with 2 mL of culture medium until maturation. In addition, the cells were also seeded into 12-well plates without a cover slip or membrane. For the cytokine experiments, pneumocytes were then exposed to 500 ng/mL of rh IL-13, -27, -31, -13 plus -31 and -13 plus -27 plus -31 for a 1 h incubation. The decided dose of these ILs was obviously seen or easily differentiated the appearance of apoptotic and normal cells without adverse effect to the cells. Additionally, for the hemozoin experiment, the pneumocytes were exposed to 20 μM of s-hemozoin for 1, 6, 12, 24 and 48 h with camptothecin-induced apoptosis as a positive control. The selected concentration could observe the apoptotic effect of hemozoin on pneumocytic cell without other interference from non-specific effect. All cells on the coverslip and membrane were primarily fixed with 2.5% glutaraldehyde in a 0.1 M sucrose phosphate buffer (SPB) (pH 7.4) for 1 h. The cells seeding onto the plate without a coverslip and membrane were trypsinized with 0.25% trypsin-EDTA and then collected for apoptotic cell staining and real-time RT (reverse transcription)-polymerase chain reaction (PCR).

The fixed cells were washed in triplicate by 0.1 M SPB, for 10 min each. Cells on the membrane were subjected to a transmission electron microscopic (TEM) study, while cells on the cover slip were stained with 0.1% trypan blue for morphological determination under a light microscope. To determine the numbers of apoptotic cells, all types of the A549 cells (e.g. mature, progenitor and apoptotic) were counted by ten high power fields (400×)/coverslip. The percentage of apoptotic cells was calculated and compared in each group.

### 7. Ultrastructural studies

#### 7.1 Scanning electron microscopy (SEM)

The previously fixed pneumocytic cells were fixed once again with 1% osmium tetroxide in 0.1 M SPB for 1 h and washed three times. The cells were then dehydrated with graded ethanol and air dried overnight. The coverslips were mounted on an aluminium stub and coated with a gold film of 20 nm thicknesses using the sputter coater (EMITECH K550, UK). All of the specimens were examined under a scanning electron microscope (JEOL JSM-6610LV, Japan) with 15 kV acceleration voltages.

#### 7.2 Transmission electron microscopy (TEM)

All lungs and A549 cells on the membrane were secondarily fixed with 1% osmium tetroxide in 0.1 M SPB for 1 h, dehydrated in graded ethanol for 10 min each, infiltrated with LR white (EMS, USA.) in 70% ethanol and then embedded in a capsule beam. The embedded lungs were polymerized in a 65°C incubator for 48 h. All blocks were cut into a 90–100 nm thickness. The ultra-thin sections were placed on a 200 square mesh copper grid. The sections were stained with uranyl acetate and lead citrate.

The fine morphology of all lung specimens and A549 cells were examined under a transmission electron microscope (Hitachi; model HT7700, Japan).To clarify the severity of malarial pigment deposition in the lung as an additional confirmation to histopathological examination, all hemozoin pigments within the alveolar area, mononuclear cell and PRBCs were counted in each grid square. In addition, all intravascular RBCs were also distinguished to either normal or infected (PRBCs) then counted and calculated to the percentage of PRBCs/grid square. Consideration to the intensity of hemozoin pigments and PRBCs, ten grid squares were evaluated for each case.

### 8 Apoptotic cells staining

To confirm the presence of apoptotic cells in pneumocytes affected by hemozoin, dual ethidium bromide (EB)/acridine orange (AO) staining was performed as described previously [27]. Following trypsinization, the non-fixed cells were washed with 0.1 M SPB, centrifuged at 3,000 rpm at 4°C for 10 min and then collected. The cell pellets were then resuspended in 50 μL of complete culture medium. The resuspended cells were stained with 100 μg/mL of EB/AO with a cell to fluorescent dye ratio of 2:1 (v/v). All of the staining procedures were performed on an ice-cold container. The pneumocytic cells were examined according to the staining pattern and cellular morphology and at least 100 cells/time point was counted using a fluorescent microscope no longer than 20 min after staining.

### 9 Real-time RT-PCR for CARD-9

To demonstrate apoptosis at the molecular level, *CARD*-9, an apoptosis regulator gene was measured in the pneumocytic cells with or without s-hemozoin-induced injury at each time point. The RNA was isolated using TRIzol® Reagent (Invitrogen, USA) in accordance with the manufacturer’s instructions. Complementary DNA synthesis and amplification were carried out in CFX96 Touch^TM^ Real-time PCR Detection system thermocycler conducted using KAPA SYBR FAST One-Step qRT-PCR Kits^®^ (KAPABIOSYSTEMS, USA) according to the manufacturer’s protocol. The following primer pairs were used: *CARD*-9; Forword 5´-CGGAATTCATGGCCGACAAGGTCCTG-3´ Reverse 5´-CGCTCGAGTTAGTCTTGCATATTAAGGTAATTTCCAGA-3´: β-actin; Forward 5´-GGCCAGGTCATCACCATT-3´ Reverse 5´-ATGTCCACGTCACACTTCATG-3´. The level of mRNA was normalized to the level of β-actin (as internal control) mRNA expression. Therefore, the relative levels of mRNA were then calculated via the 2^-ΔΔCt^ method.

### 10 Statistical analysis

All data were statistically analysed using the GraphPadPrism^®^ program, version 6.05. A non-parametric one or two-way analysis of variance (ANOVA) with multiple comparisons, an unpaired t-test and a Spearman’s correlation analysis were used for determining the differences between the groups and the correlation between factors of interest. The significance level was determined at a threshold of *p* < 0.05.

## Results

### 1. Clinical data

The clinical data, complications and histopathological changes in each group of patients are summarized in Table 1. In the present study, the ages of the patients with severe malaria (SM) range between 3–54 years of age. There was no difference in the ages between the ARDS and non-ARDS groups. The level of parasitemia at the time of admission in both groups was extremely high; however, a statistical difference was not found. Regarding the clinical and histopathological appearance, 31.03% (9/29) of the SM patients presented with signs of ARDS. The occurrence of PE and systemic bleeding in ARDS patients was significantly higher than those in non-ARDS patients. In addition, the non-ARDS patients had a significantly higher occurrence of acute renal failure than the ARDS group. However, the ARDS patients presented with a similar occurrence of anaemia, jaundice, acidosis, shock, hypoglycaemia and cerebral malaria when compared to the non-ARDS patients. Moreover, some positive correlations between clinical observations and histopathological findings were also found. The higher score of alveolar expansion related to the presence of acidosis (Spearman’s rho = 0.498, p = 0.006). The increased level of pulmonary malarial pigment deposition also related to anaemia (Spearman’s rho = 0.424, p = 0.022). Finally, high density of PRBCs and WBCs accumulations correlated to the incidence of pneumonia (Spearman’s rho = 0.543, p = 0.002 and Spearman’s rho = 0.417, p = 0.024, respectively).

**Table 1:** Comparison of the clinical data, histopathological changes and clinical complications in severe malaria cases with and without ARDS.

Parameter	Non-ARDS	ARDS	p-value
**Clinical data and complications**			
Age (year)	3–54 (25.0 ± 15.8)	15–35 (22.7 ± 6.1)	0.690
Parasitaemia/μL upon admission	606,595.50 ± 778,259.56	393,787.77 ± 470,155.51	0.456
Anaemia (%)	75.0 (15/20)	55.6 (5/9)	0.266
Jaundice (%)	75.0 (15/20)	77.8 (7/9)	0.631
Pneumonia (%)	25.0 (5/20)	77.8 (7/9)	0.012
Acute renal failure (%)	45.0 (9/20)	0.0 (0/9)	0.017
Acidosis (%)	35.0 (7/20)	33.3 (3/9)	0.636
Shock (%)	15.0 (3/20)	22.2 (2/9)	0.502
Systemic bleeding (%)	0.0 (0/20)	55.6 (5/9)	0.001
Hypoglycaemia (%)	10.0 (2/20)	11.1 (1/9)	0.688
Cerebral malaria (%)	70.0 (14/20)	55.6 (5/9)	0.364
**Quantitative lung ultrastructure**			
% PRBCs sequestration	55.5 ± 40.34	75.0 ± 36.09	0.206
Malarial pigment/grid square	10.8 ± 6.63	22.6 ± 11.76	0.002
**Histopathological changes (median score)**			
Pulmonary oedema (%)	65 (13/20)	77.8 (7/9)	0.041
Alveolar expansion	1.63 ± 1.11	1.40 ± 0.84	0.540
Septal congestion	1.78 ± 0.92	1.70 ± 0.48	0.776
Malarial pigment	1.75 ± 0.93	2.40 ± 0.84	0.040
Alveolar haemorrhage	0.69 ± 0.98	2.00 ± 0.94	0.001
PRBCs sequestration	1.48 ± 1.06	1.30 ± 0.82	0.617
WBC accumulation	2.03 ± 0.72	2.20 ±0.42	0.489
Hyaline membrane formation	0.0 ± 0.0	1.30 ± 0.67	0.000

Note; ARDS; acute respiratory distress syndrome, PRBS; parasitized red blood cells, WBC; white blood cell, %; percent, μL; microlitter

### 2. Histopathology

**Fig 1:**
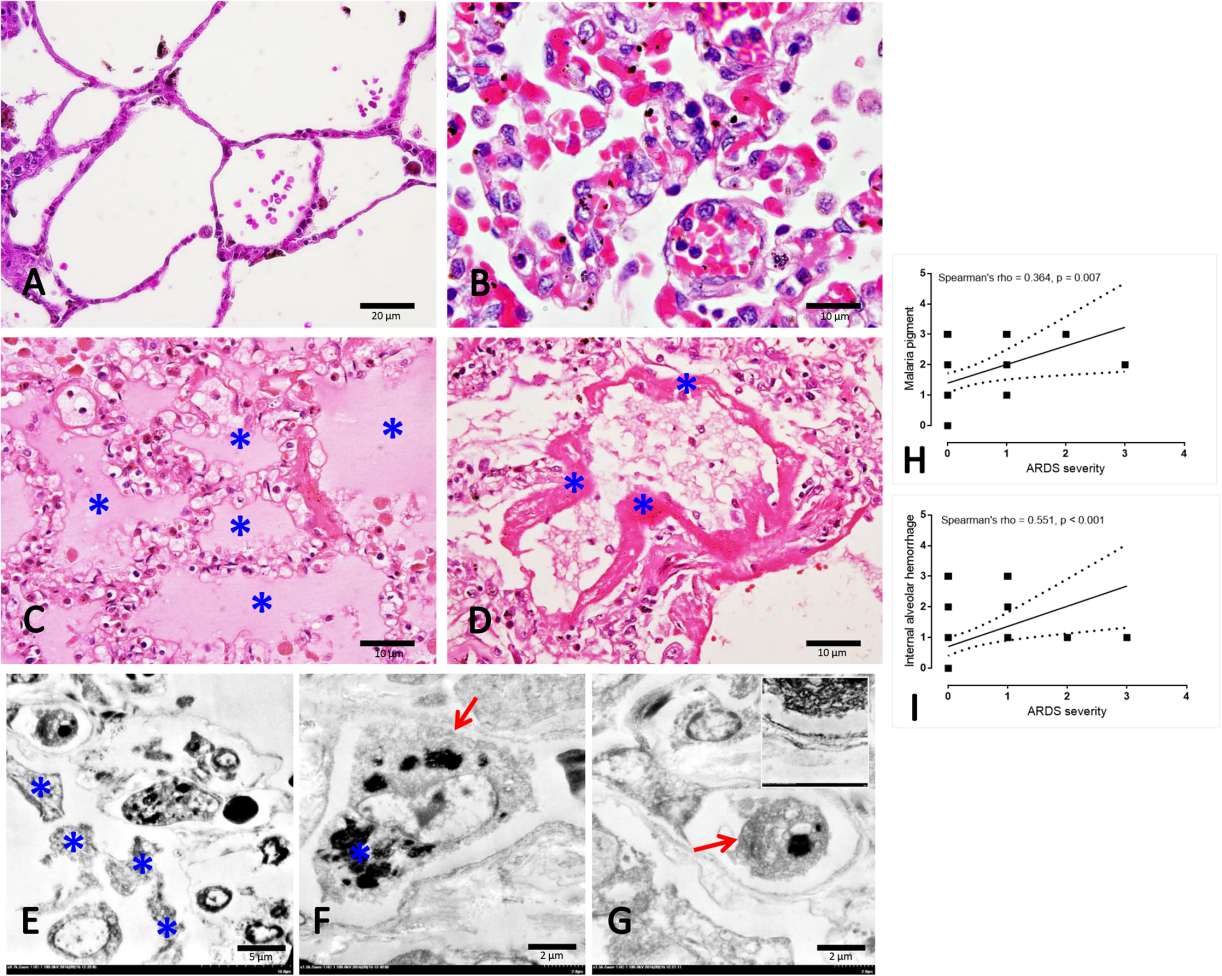
Histopathological and ultrastructural appearance in normal and severe malarial lungs. A: normal lung represented by a very thin alveolar septum and clearly defined alveolar sac in contrast to the non-PE lung (B) which was characterized by thickened alveolar septum congested with blood components (e.g. PRBCs and WBCs). Apart from the thickened membrane, fluid (C*) and the hyaline membrane (D*) were deposited in the PE and ARDS lungs, respectively. Fine morphology of hyaline membrane (E*), macrophage (F; arrow)-ladened hemozoin pigment (F*) and PRBCs (G; arrow) with endothelial cell damage (G-inset) were frequently observed in the ARDS lung patients. A positive correlation between some histopathological changes and ARDS severity was calculated using Spearman test (H-I).

Regarding the pulmonary complications in the subjects with falciparum malaria, the lung histology was altered, including alveolar septal thickening and congestion accompanied by the deposition of cellular and blood components. In particular, PRBCs and WBCs (Fig 1B) were found in the fluid contained within the alveoli (Fig 1C) and alveolar epithelium damage associated with hyaline membrane formation (Fig 1D) was also related to the widespread inflammation. The histopathological scoring revealed that in ARDS patients, scores for malarial pigment, alveolar haemorrhage and hyaline membrane formation were significant compared to those in patients without ARDS (Table 1). In the present study, from 9 patients who had ARDS, 77.8% (7/9) of them had PE. The severity of PE was examined, approximately seventy percent (71.42%; 5/7) of ARDS patients with PE showed the presence of oedematous fluid in the alveolar sac between 25–50% of the section while 28.57% (2/7) had severe score of PE.

### 3. Lung ultrastructural study

The fine morphological structure of the ARDS lungs was predominantly characterized by a discontinuation of the alveolar membrane, the formation of a hyaline membrane within the alveolar space, generalized endothelial cell degeneration and a high number of macrophages laden with malarial pigment (Fig 1E–1G). It was noted that there were rarely type 2 pneumocytic cells present in the ARDS patient lungs. Moreover, ARDS severity was correlated significantly with the presence of malarial pigment and internal alveolar haemorrhaging (Fig 1H and 1I).

### 4. Immunohistochemistry and histochemistry

#### 4.1. EPCR, TM, ICAM-1 and TNF-α in ARDS and non-ARDS lungs

**Fig 2:**
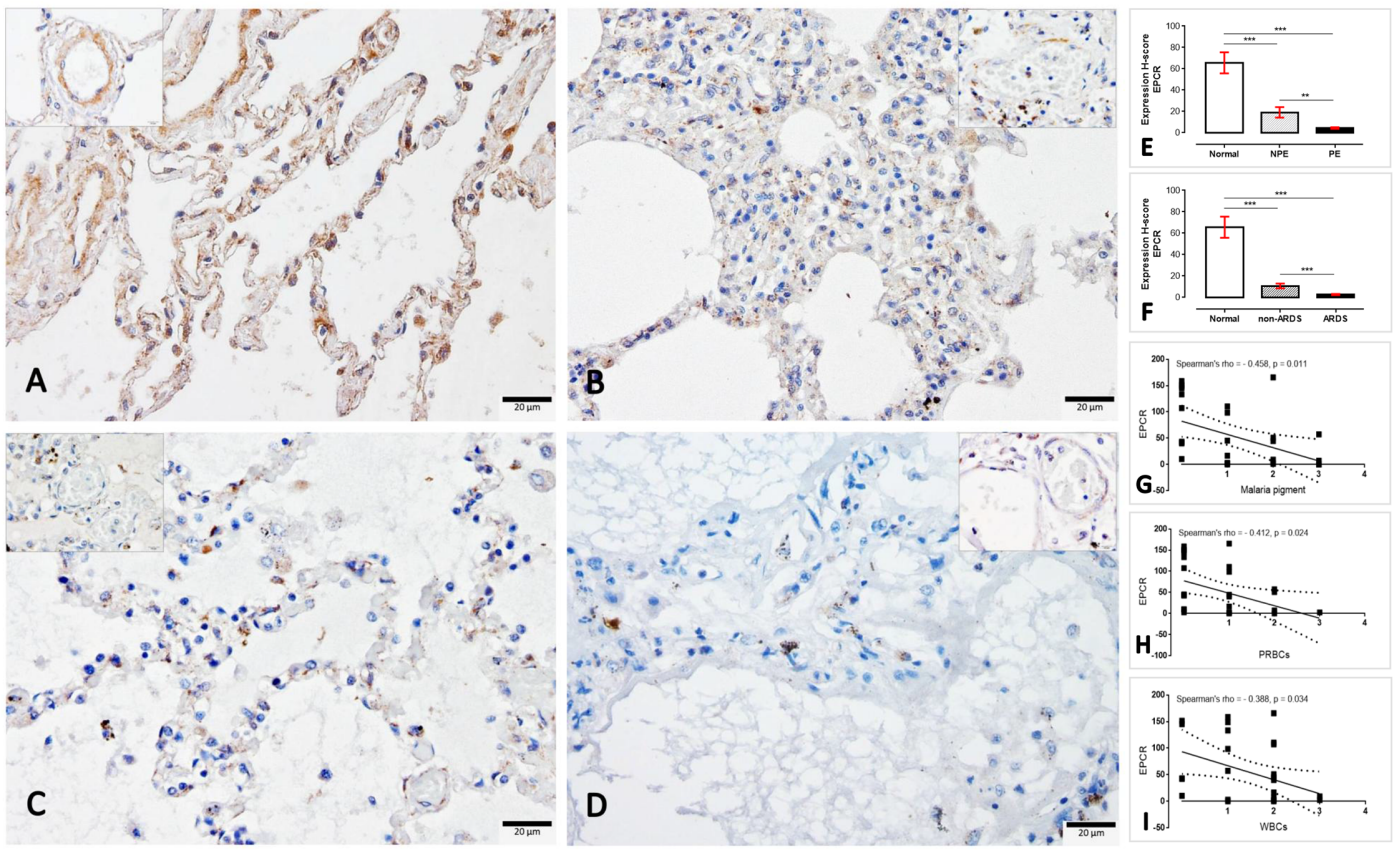
**Immunohistochemical staining of EPCR in the normal (A), non-PE (B), PE (C) and ARDS (D) lungs**. The EPCR expression in small blood vessels are shown in the inset of each micrograph. The H-score expression is demonstrated by bar graphs for PE (E) or ARDS (F) lungs, to compare those expression Friedman test was performed (**; p-value < 0.001, ***; p-value <0.0001). Negative correlation between the level of EPCR and some histopathological changes was observed using Spearman test (G-I).

To determine the expression of EPCR, TM, ICAM-1 and TNF-α in response to the extent of lung injury in SM patients with and without ARDS, immunohistochemical staining was performed. Similarly, EPCR and TM were expressed on endothelial cells as well as on the tunica medial layer, alveolar septum and pneumocytic cells. Moreover, ICAM-1 and TNF-α were also primarily expressed on the endothelial cells and sparsely on mononuclear cells. In the present study, the expression score was considered for the immune-positive stained areas in alveolar septa. The semi-quantitative H-score demonstrated that the EPCR in PE lungs had a significantly lower expression when compared to non-PE and normal lungs, respectively (Fig 2). Regarding the ARDS category, ARDS lungs also exhibited a significantly lower expression of EPCR than those from non-ARDS and normal subjects, respectively. Interestingly, there was a negative correlation between the EPCR level and the malarial pigment, PRBC and WBC deposition in the lung (Fig 2G–2I).

**Fig 3:**
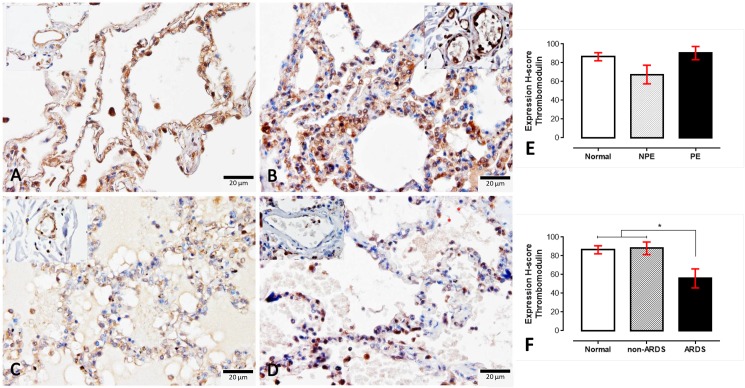
**Immunohistochemical staining for thrombomodulin in normal (A), non-PE (B), PE (C) and ARDS (D) lungs**. The level of thrombomodulin expression in the small blood vessels are shown in the inset of each micrograph, The H-score expression is demonstrated by bar graphs for PE (E) or ARDS (F) lungs, to compare those expressions Friedman test was performed (*; p-value < 0.05).

**Fig 4:**
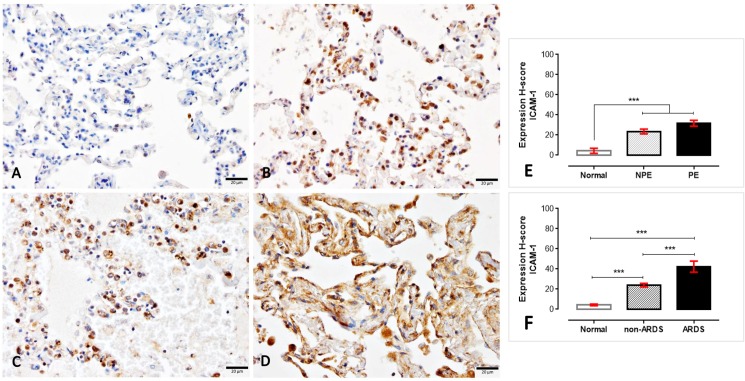
I**mmunohistochemical staining for ICAM-1 thrombomodulin in normal (A), non-PE (B), PE (C) and ARDS (D) lungs**. The H-score expression is demonstrated by bar graphs for PE (E) or ARDS (F) lungs, to compare those expression Friedman test was performed (***; p-value <0.0001).

**Fig 5:**
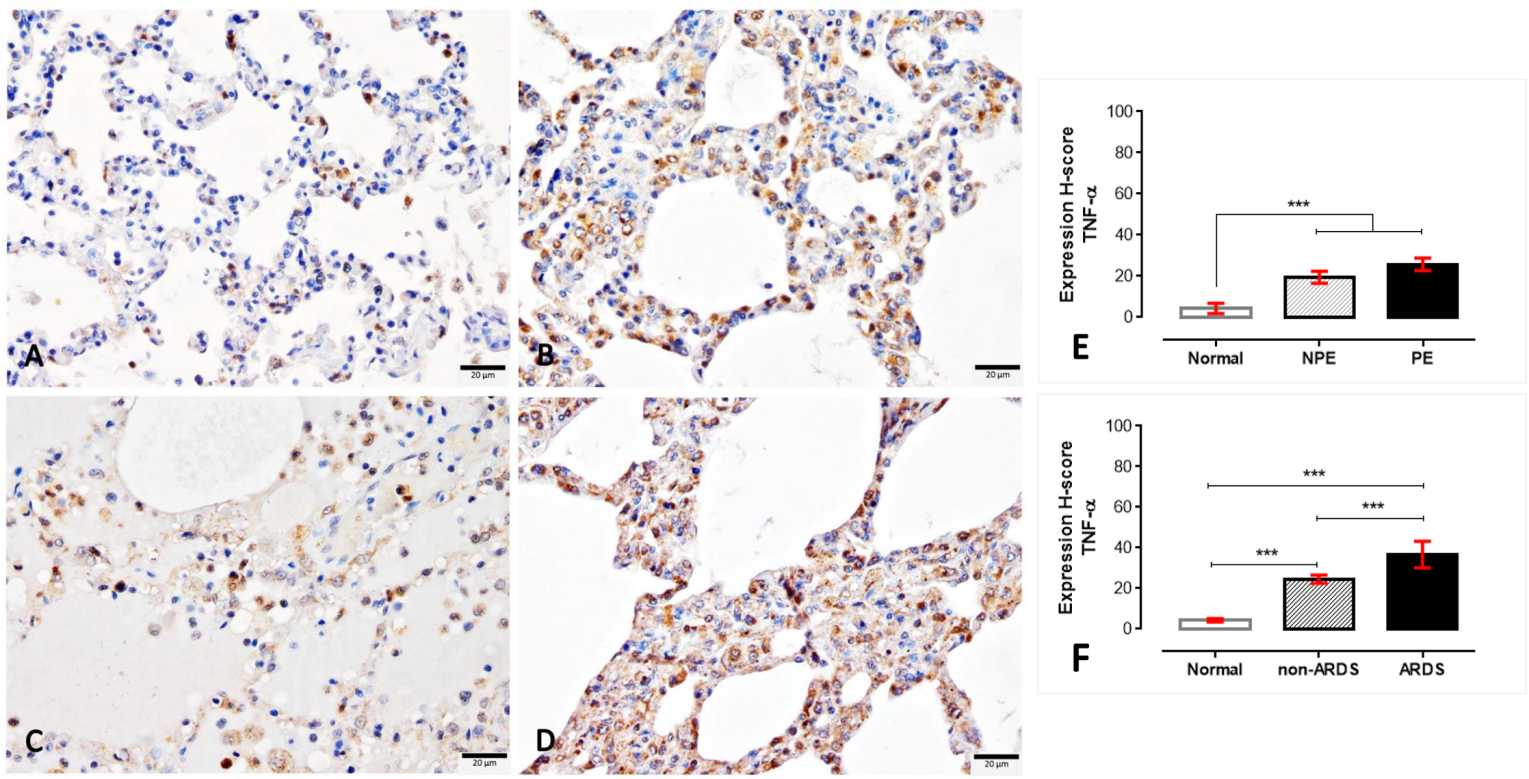
I**mmunohistochemical staining for TNF-α in normal (A), non-PE (B), PE (C) and ARDS (D) lungs**. The H-score expression is demonstrated by bar graphs for PE (E) or ARDS (F) lungs, to compare those expression Friedman test was performed (***; p-value <0.0001).

Although there were no differences found regarding the expression of TM on normal, non-PE and PE lungs, the ARDS lungs had a lower expression of TM compared to normal and non-ARDS lungs (Fig 3). In contrast with TM expression, ICAM-1 was strongly labelled on the ARDS and PE lungs compared to the non-ARDS and non-PE lungs (Fig 4). Like ICAM-1, TNF-α also significantly higher expressed in SM patients than normal subjects (Fig 5). With the similar pattern of ICAM-1 expression, SM patients with ARDS had significantly higher levels of TNF-α when compared to SM patients without ARDS and normal subjects, respectively.

#### 4.2. Intravascular fibrin in ARDS and non-ARDS lungs

**Fig 6:**
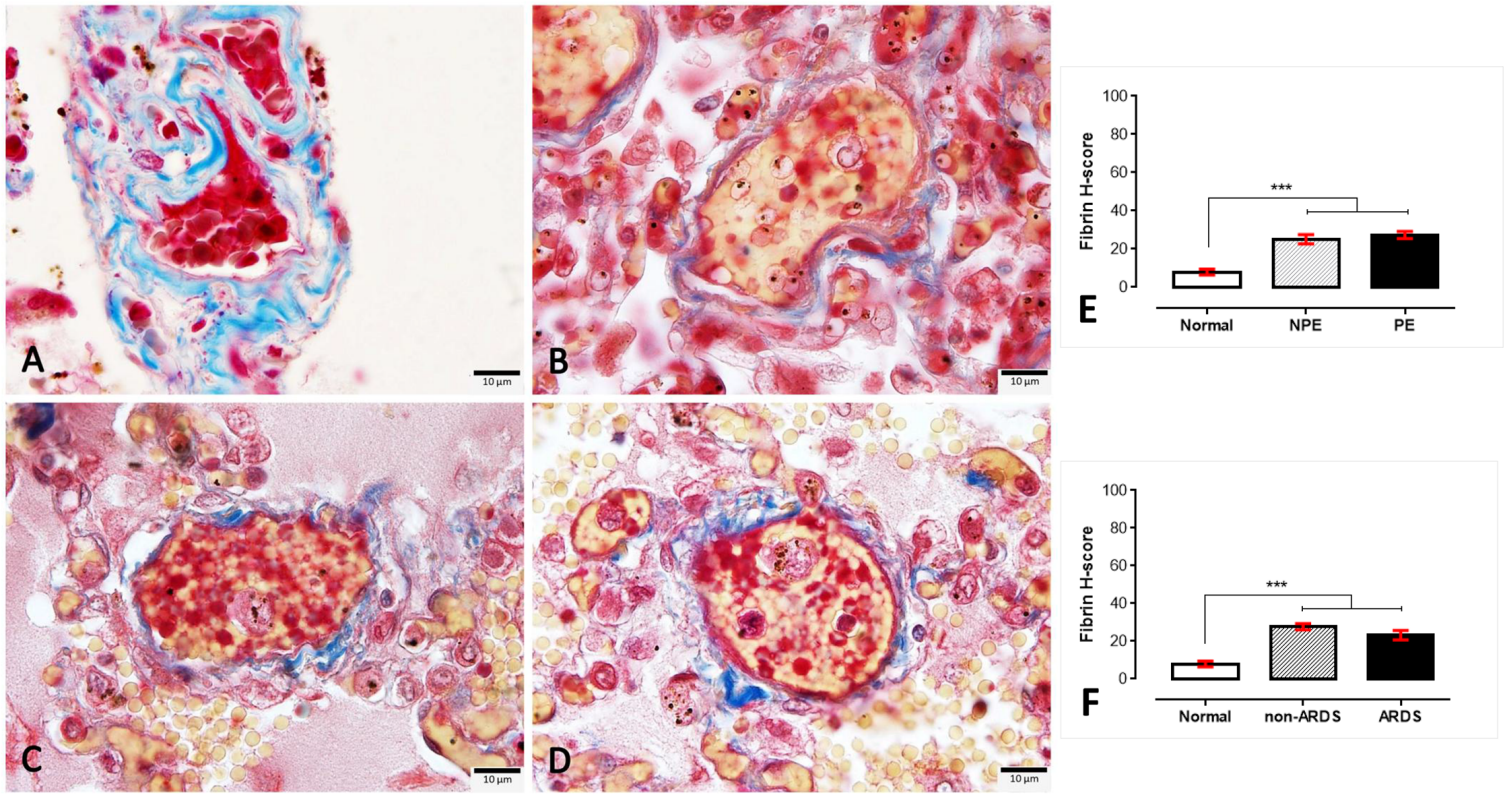
**Masson’s trichrome staining for intravascular fibrin deposition in normal (A), non-PE (B), PE (C) and ARDS (D) lungs**. The fibrin staining is exhibited in the yellowish material found in the small blood vessels, whereas red blood cells and collagen fibrils were stained red and blue, respectively. The H-score expression is demonstrated by bar graphs for PE (E) or ARDS (F) lungs, to compare those expression Friedman test was performed (***; p-value <0.0001).

In the present study, fibrin deposition in the small blood vessels was evaluated to define the coagulation abnormalities in the lungs of SM patients with and without PE or ARDS. The amount of intravascular fibrin was very high in SM patients when compared to normal lungs; however, there was no significant difference in the level of fibrin deposition between PE and non-PE or ARDS and non-ARDS lungs (Fig 6).

### 5. Cytokine-induced A549 cell injury

**Fig 7:**
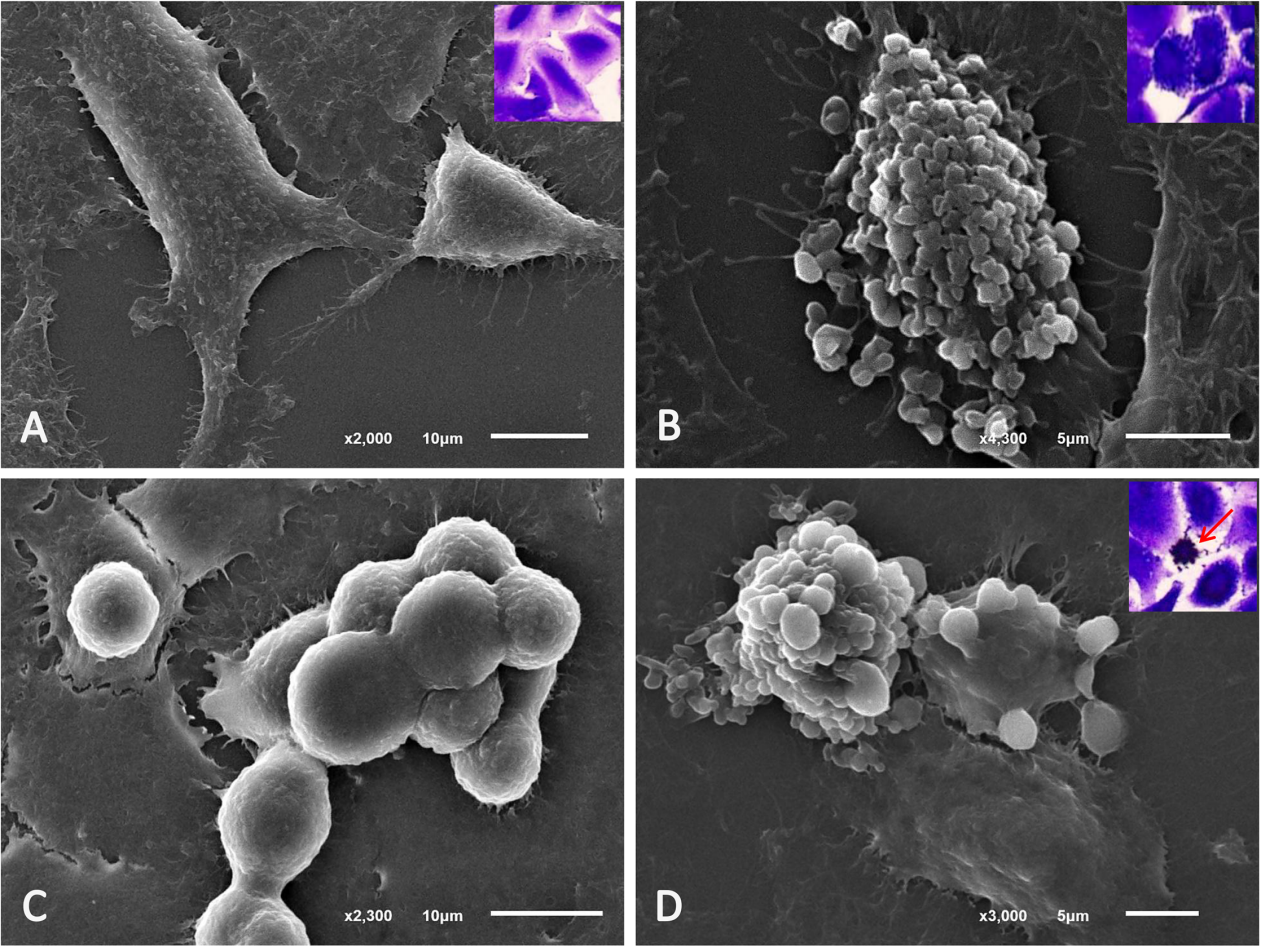
Scanning electron micrographs of A549 cells. A549 cell morphology was composed of many cell types: 1) the mature form with (A) and without granules (B) were the largest cells with an irregular and granulate surface; 2) the progenitor form consisted of small and rounded cells with a smooth and irregular membrane (C) and 3) apoptotic cells (D) are the shrunken cells with numerous vacuolated membranes. Cells stained with trypan blue cells were shown in the inset of each micrograph.

**Fig 8:**
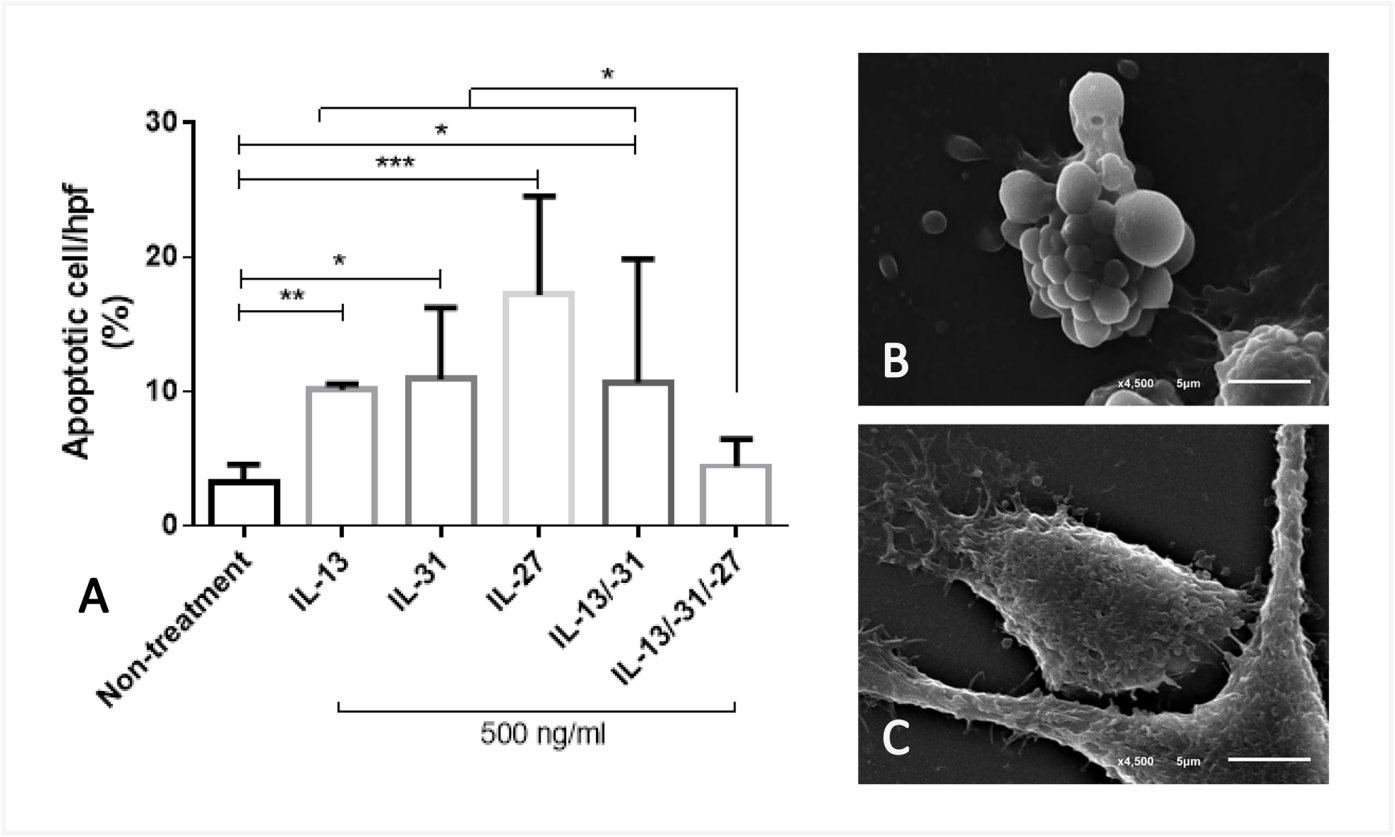
Percentage of apoptotic cells induced by cytokines. The bar graph exhibited the difference in the number of apoptotic cells in each group using Friedman test (*; p-value<0.05, **; p-value <0.001, ***; p-value<0.001) (A) which was morphologically characterised by apoptotic (B) and normal cells (C).

In relation to the cytokine-induced lung injury, recombinant human (rh) IL-13, -31 and -27 were chosen as the representative cytokines that are frequently present in SM, to determine their effect on lung epithelial cells. In general, using a scanning electron microscopy, an A549 cell is a pneumocytic cell that exhibits three typical morphologies, including: mature, progenitor and apoptotic cell types (Fig 7). Co-culturing the A549cells with these cytokines at a dosage of 500 ng/ml demonstrated that all molecules applied to the lung epithelial cells resulted in a significantly increased number of apoptotic cells when compared to the untreated group (Fig 8). Interestingly, when the combination of IL-27, IL-13 and IL-31 was added to the A549 cell, the number of apoptotic cells declined when compared to all examined cytokines alone.

### 6. S-hemozoin-induced A549 cell injury through an apoptotic pathway

**Fig 9:**
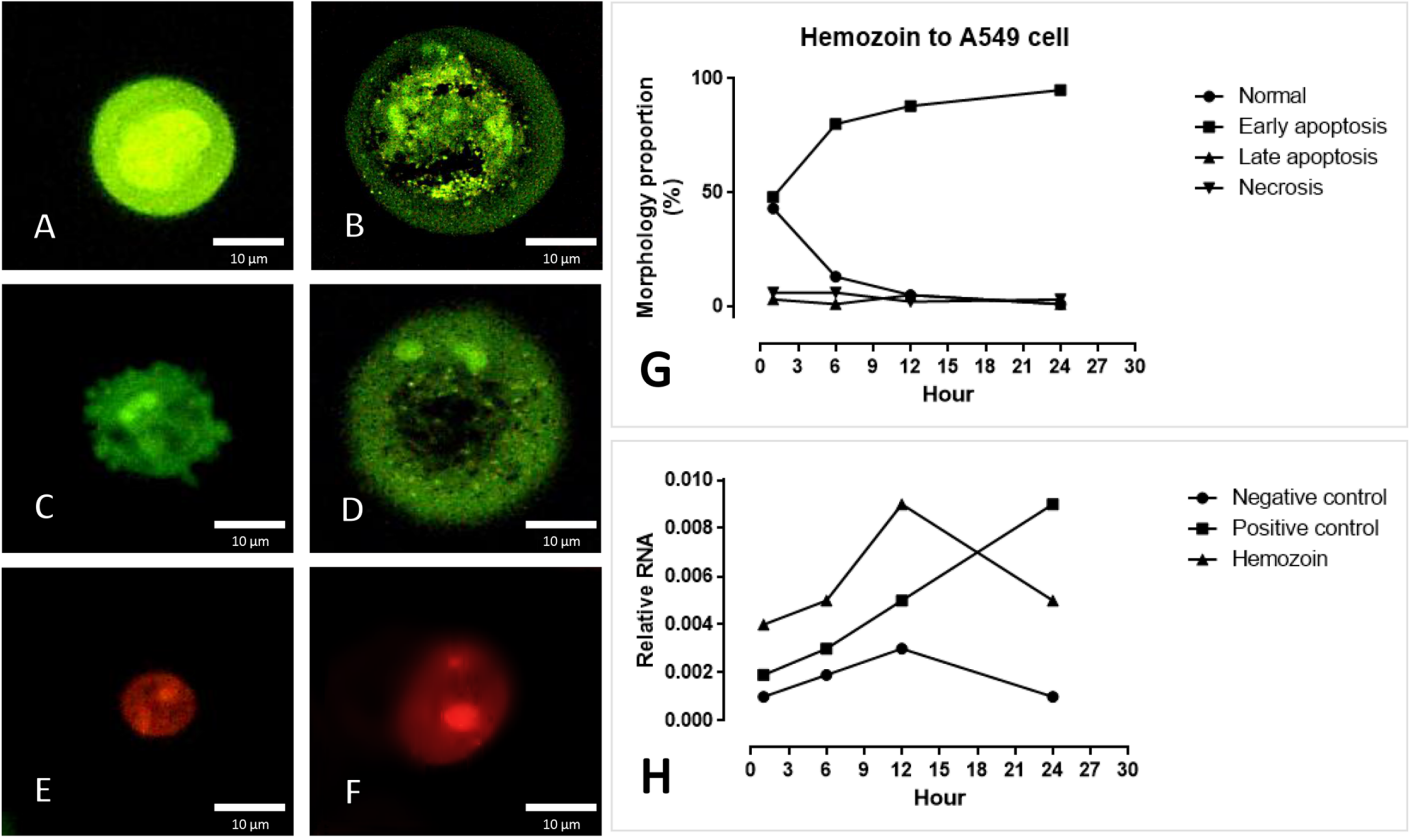
Apoptosis indicated by EB/AO staining and real-time RT-PCR. S-hemozoin-induced pneumocytic apoptosis was characterized by morphological changes and staining patterns that included normal cells (A), early apoptotic cells with nuclear fragmentation or condense chromatin (B), membrane blebbing (C), cytoplasmic vacuolization (D), late apoptotic cells with condensed chromatin (E) and necrotic cells (F). The bar graph demonstrates that early apoptosis was predominately found following hemozoin treatment between 1–24 h with an increasing trend (G) in accordance with the mRNA expression of *CARD*-9 (H).

**Fig 10:**
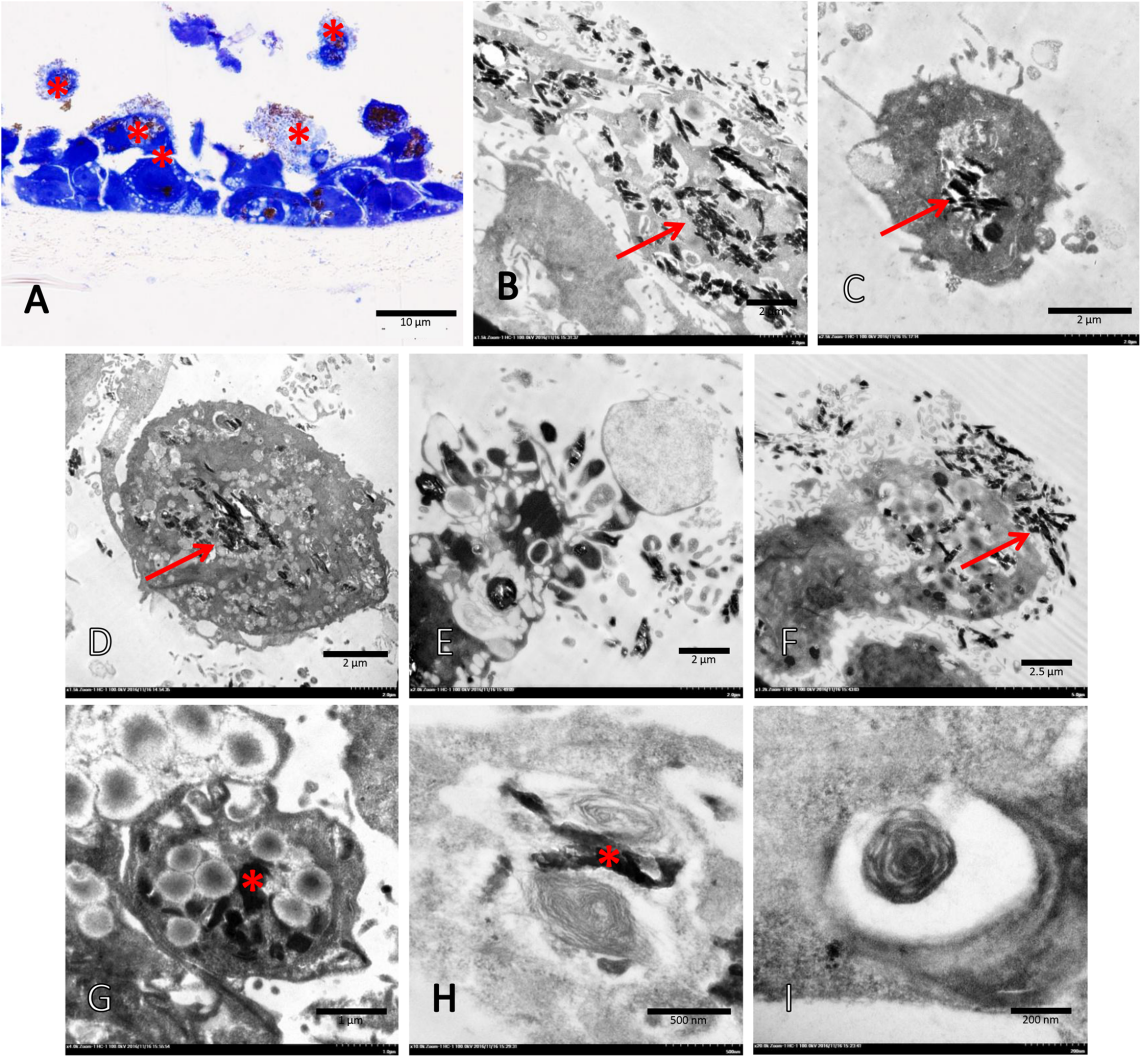
Transmission electron micrograph of A459 cells exposed to s-hemozoin for 24 h. A semi-thin section of A549 cells was characterized by toluidine blue staining, including normal cells with or without hemozoin, that adhered to the membrane and apoptotic cells (*) with hemozoin, which slipped out of the membrane (A). A number of hemozoin pigments (arrow) were ingested by intact (B-C) and apoptotic (E-F) A549 cells, were located in multivesicular (G) or lamellar (H-I) bodies characterized by dark electron dense material (*).

To identify the apoptotic effect of s-hemozoin on pneumocytic cells, dual EB/AO staining and real-time RT-PCR were performed. The results revealed that s-hemozoin itself induced early apoptosis predominantly in lung epithelial cells as characterised by their morphological changes and staining patterns. Briefly, when compared to normal cells (Fig 9A) with an intact nuclear and cytoplasmic membrane, early apoptotic cells were shown in green fluorescence with nuclear fragmentation or condensed chromatin (Fig 9B), cellular blebbing (Fig 9C) and cytoplasmic vacuolization (Fig 9D). Late apoptotic cells exhibited orange-red fluorescence with condensed chromatin (Fig 9E), whereas the necrotic cells were stained fluorescent red with an intact nucleus (Fig 9F). Similar to the EB/AO staining results, a higher level of *CARD*-9 was observed in the cells treated with hemozoin for 1, 6 and 12 h compared to the negative control with a similar pattern (Fig 9H). However, 24 h post-treatment, *CARD*-9 gene expression was diminished in both groups in contrast with the positive control. Interestingly, the ultrastructural analysis revealed that the A549 cells were predominantly pneumocyte type II cells, appeared to have phagocytic activity and ingested hemozoin pigment in our *in vitro* conditions (Fig 10). At 24 h post-culture, the apoptotic pneumocytes presented as detached cells separated from the membrane with cytoplasmic membrane blebling (Fig 10E). Ingested hemozoin was mainly found in surfactant contained cells as presented by immature cells or multi-vesicular bodies (rounded and dense packed vesicle) (Fig 10G) and mature cells or lamellar bodies (electron dense lamellate structyre) (Fig 10H and 10I).

## Discussion

The present study found that malaria-associated ARDS was significantly associated with PE and systemic bleeding (Table 1). Moreover, a positive correlation between internal alveolar haemorrhage and the severity of ARDS was observed (Fig 1). Generally in SM, systemic bleeding is caused by a coagulation disturbance which consumes clotting factors and leads to severe bleeding in various tissues [10]. For this reason, several studies have focused on the correlation between coagulation abnormalities and SM. Interestingly, our results revealed that the severity of ARDS was dependent on the level of malarial pigment deposited in the lungs of SM patients (Fig 1). Therefore, our findings suggest that the alteration of the EPCR and TM in association with the deposition of s-hemozoin in the lung, including their interactions, may play a role in the pathogenesis of ARDS in SM.

Throughout the past decade, the protein C system had been considered a potentially important factor in the pathogenesis of SM. The early and preventive using of activated protein C as an adjunctive treatment for falciparum malaria and leptospirosis co-infection leads to improved clinical symptoms, particularly of lung injury, due to its anti-thrombotic, pro-fibrinolytic and anti-inflammatory properties [28]. Endothelial protein C reacts with parasitic materials from ruptured PRBCs and reduces their pro-inflammatory effects on endothelial cells [29]. Recently, PfEMP-1, the parasitic ligand on PRBCs infected with *P*. *falciparum*, has been shown to adhere to the EPCR on host cells and to interfere with the anti-coagulant and pro-endothelial barrier functions, which leads to activated coagulation, enhanced tumour necrosis factor (TNF) production and increased vascular permeability. All of these factors contribute to the pathogenesis of cerebral malaria [18, 30–32]. Our immunohistochemistry experiment revealed that a reduction of EPCR and TM was predominantly found in SM patients with ARDS (Figs 2 and 3). PE lungs also exhibited a lower expression of EPCR than those from non-PE and normal subjects, respectively. Fibrin was highly deposited inside the pulmonary vessels of SM patients with or without ARDS (Fig 6). There was a negative correlation between EPCR expression and the level of s-hemozoin, PRBC and WBC deposition in the lungs (Fig 2). Furthermore, ARDS lungs had a significantly higher level of ICAM-1 and TNF-α expression than the non-ARDS lungs, whereas there was a similar level of ICAM-1 expression in both PE and non-PE lungs (Fig 4). SM patients with ARDS experienced a loss of EPCR and thrombomodulin expression in association with a high level of intravascular fibrin, hemozoin deposition, PRBC sequestration and inflammatory activation. In addition, the present study also indicated that the increase of ICAM-1 and TNF-α expression was often found together with the decrease of TM in the lung. This relevance is in agreement with previous studies [33, 34] due to their antagonistic effect of ICAM-1, and TNF-α and TM in term of the increment of vascular permeability and vascular protective property, respectively. To the best of our knowledge, our report describes that the pathogenesis of ARDS is associated with the protein C system accompanied by hemozoin accumulation in the lung.

Cytokine activation is one of the main contributors of pathogenesis in SM affecting pulmonary epithelial and endothelial cells, leading to acute lung injury or ARDS. To predict severity in SM, chemokine and cytokine production may be useful for diagnosis and prognosis. As shown in the present study, a culture of IL-27 combined with IL-13 and IL-31 alleviated the number of apoptotic cells among the pneumocytes within 1 h when compared to those of each cytokine alone (Fig 8). The findings of the present study demonstrated that acute lung injury was related to circulatory pro- and anti-inflammatory cytokines presented in SM. However the result also presented that IL-27 alone induced a high number of pneumocytic cell apoptosis. It is probably caused by some interaction between immune-modulatic role of IL-27 and carcinogenicity of A549 cell. In general, IL-27 modulates immunologic function that expresses by either proinflammatory or anti-inflammatory effects. In cancer cells, IL-27 can activate apoptosis via several pathways as presented in some reports [35, 36]. A549 cell is derived from adenocarcinoma origin. Because of its carcinogenicity, IL-27 may react to this cell as proinflammatory effect and lead to apoptosis.

It has been well established that the decrease in pulmonary surfactant produced by type II pneumocytes plays a crucial role in malaria-associated ARDS pathogenesis. In addition, another possible hypothesis is the multi-factorial nature of the pro-oxidative stress environment, including: [37] 1) the increase of polyunsaturated fatty acids due to the degradation of oxidative lipids; 2) proteolytic enzymes and pro-inflammatory cytokines from the host inflammatory responses [38, 39] and 3) the presence of hemozoin and haemoglobin degradation waste products from the parasite [40]. All of these factors may contribute to acute lung injury, including blood gas barrier leakage, haemorrhage and alveolar oedema. Consistent with previous reports in both experimental models [40, 41] and human cases [42], our results revealed that pulmonary hemozoin deposition was significantly increased in ARDS lungs in conjunction with PE and systemic bleeding (Table 1). Moreover, a positive correlation between the level of hemozoin and ARDS severity was observed (Fig 1H). Importantly, the results revealed that the deposited pigment also exhibited a negative correlation with the expression of EPCR in the lungs (Fig 2G), which was associated with the alteration of pulmonary epithelial integrity and vascular permeability. Therefore, the present study indicated that the level of hemozoin may be an important factor related to the occurrence of ARDS in patients with severe malaria.

In addition to studies in malaria-associated ARDS patients, a culture of pneumocyte and s-hemozoin was conducted in this study. Our results indicated that s-hemozoin caused pneumocytic cell injury and apoptosis as exhibited by EB/AO staining and *CARD*-9 expression (Fig 9). Concerning the onset of apoptosis, within 24 h post-exposure, s-hemozoin induced early apoptosis in pneumocytes as characterized by their staining patterns and morphological changes (Fig 9A–9G). In agreement with the mRNA expression of *CARD*-9, apoptosis was presented within 12 h post-exposure with an increasing trend (Fig 9H). Thus, it is suggested that in addition to the pro-inflammatory effects induced by hemozoin, alveolar epithelial cell damage was directly caused by s-hemozoin due to its pro-oxidative properties [40] via an apoptotic pathway. Recently, an *in vitro* study of *P*. *falciparum*, isolates demonstrated that apoptosis in pulmonary endothelial cells is sensitive to oxidative environments and requires direct contact between the parasite and host cells, otherwise PRBCs are sequestered alone [43]. Therefore, the oxidative properties of hemozoin may destroy the pulmonary epithelium and lead to acute lung injury.

Fas/FasL has been well described as pro-apoptotic signal, which is implicated in ARDS pathogenesis due to caspase activation [44, 45]. Interestingly, not only apoptotic genes those related to malaria-associated lung injury e.g. plasmodium apoptosis-linked pathogenicity factors (PALPF) [43] and caspase-3 and -8 [46, 47], Fas/FasL also contributes to both lung endothelium and epithelium damages leading to PE [47]. However, to date, there have been no reports of apoptotic-associated ARDS in severe falciparum malaria patients. The present study utilized an *in vitro* culture system to demonstrate lung epithelial cell damage and apoptosis in response to s-hemozoin as shown by *CARD*-9 expression. *CARD*-9 is the regulator of the apoptotic process through the nuclear factor (NF)-κB signalling [48]. This signalling modulates apoptosis in brain endothelial cell, leukocyte and hepatocyte in severe falciparum malaria patients [49, 50]. Consequently, it has a high possibility that *CARD-9* involves in pulmonary apoptotic activity in severe malaria as seen in the brain and liver. Recently *CARD*-9 was also found to relate to the severity of lung injury leading to ARDS due to its activating effect on inflammatory responses [51, 52]. Therefore, the up-regulation of *CARD-9* in pneumocytes treated with s-hemozoin may correspond with the level of lung injury due to apoptotic activity.

**Fig 11:**
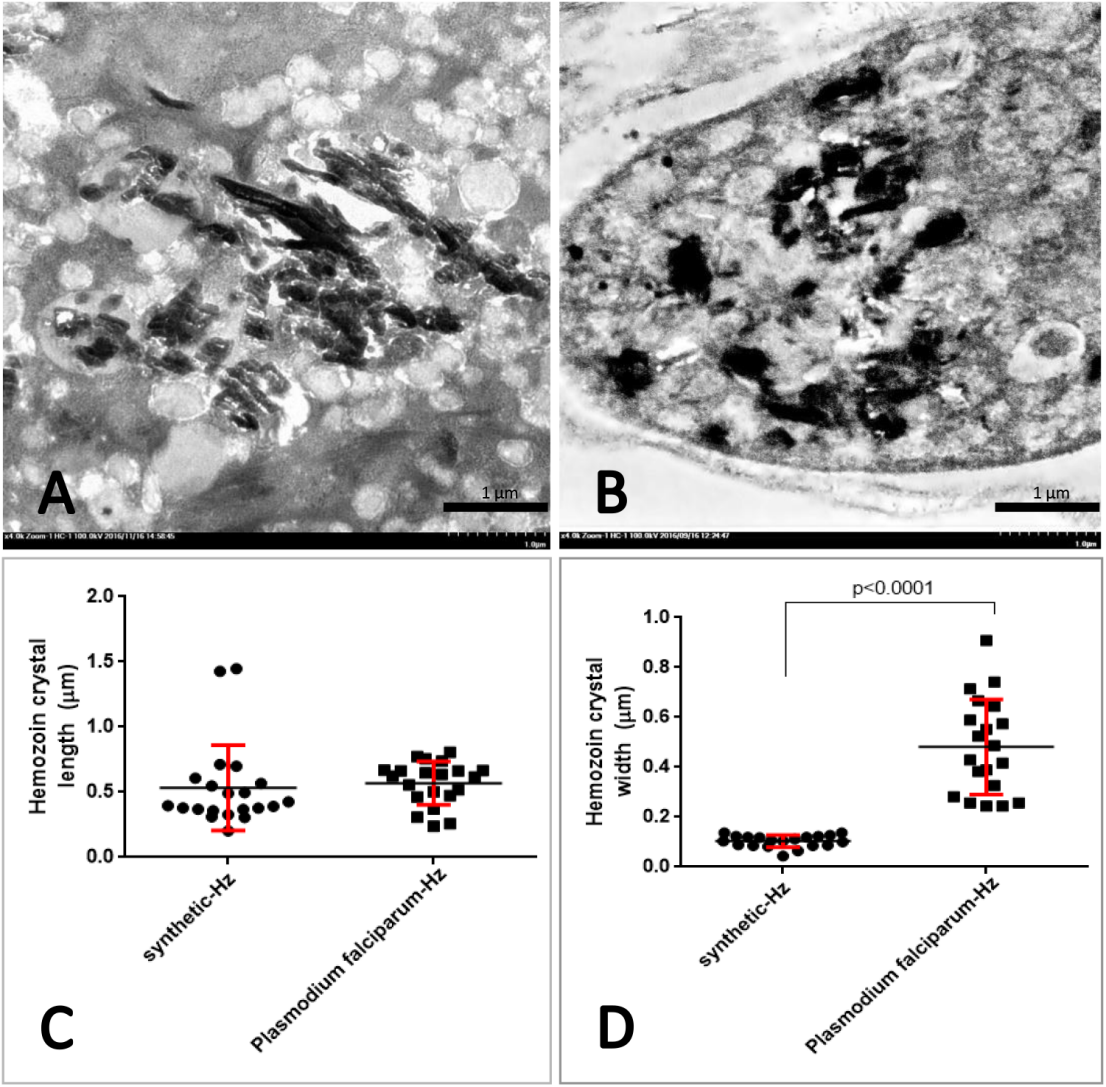
Size comparison of synthetic and *P*. *falciparum* hemozoins. Ultrastructural micrographs of the s-hemozoin in the A549 cell (A) and Pf-hemozoin in the mononuclear cell (B). The dot graph exhibited the difference in length (C) and width (D) of hemozoin crystal using Mann-Whitney test.

Several studies have reported that A549 cells exhibit phagocytic activity in association with host defence mechanisms such activity can be induced by foreign materials, fungi, or bacteria [53–55]. A transmission electron microscopic study revealed that A549 cells, predominantly pneumocyte type 2 cells, ingested s-hemozoin 24 h post-exposure in conjunction with the presence of injured cells undergoing apoptosis and necrosis (Fig 10). S-hemozoin was primarily internalised in A549 cells and located within the lamellar bodies, specialised vesicles for surfactant phospholipid deposition. However, the details of the phagocytic mechanism of these cells require further study. In addition, it has been well established that the source of vascular and pulmonary hemozoin in SM cases is the ruptured and mature PRBCs which are typically phagocytosed by neutrophils, monocytes and macrophages [6]. Our *in vitro* study proposed one more hypothesis, namely that the pneumocyte type 2 cells may interact with hemozoin and play an important role in the acute lung injury associated with SM as demonstrated by their phagocytic properties. However, our *in vitro* study had its own limitation, especially by the properties of s-hemozoin when compared to Pf-hemozoin from SM patients. S-hemozoin with acidic synthesis tends to be contaminated with DNA, depending on its purity of used hemin, and causes a huge consequence on the bio-cellular activities [56]. Moreover, a different type of hemozoin especially in size leads to their dissimilarity properties. As our observation, although the length of both types of hemozoin was similar, Pf-hemozoin had significantly wider than presented in s-hemozoin (Fig 11). Moreover, the shape of s-hemozoin presented in the pneumocyte seemed to be elongated crystalline, while a clumped form was mainly observed in Pf-hemozoin from malarial lung specimens. This kind of difference may affect on the level of cellular responses linking to the mimic ability of *in vitro* system to *in vivo* situation. To clarify this point, further studies need to be proven with better concerning of both size and purity of the used hemozoin.

## Conclusions

In severe malaria-associate ARDS, anti-coagulation properties, characterized by the EPCRs and TM, and as well as pulmonary integrity are impaired. In conjunction with pro-inflammatory cytokines and hemozoin deposition, the type II pulmonary epithelium is destroyed through the apoptotic pathway as confirmed by the up-regulation of *CARD*-9. Therefore, the pulmonary resolution is blocked and the blood gas barrier is further disrupted and finally leads to an accumulation of fluid in the alveolar space accompanied by hyaline membrane formation. The inhibition of any point along this pathway is considered to be a beneficial novel candidate for adjunctive therapy of severe malaria-associated ARDS.

## References

[1] WHO. World malaria report: 2013. Switzerland: World Health Organization; 2013.

[2] Aursudkij B, Wilairatana P, Vannaphan S, Walsh DS, Gordeux VR, Looareesuwan S. Pulmonary edema in cerebral malaria patients in Thailand. Southeast Asian J Trop Med Public Health. 1998;29(3):541–5. 10437953

[3] Bruneel F, Hocqueloux L, Alberti C, Wolff M, Chevret S, Bedos JP, et al The clinical spectrum of severe imported falciparum malaria in the intensive care unit: report of 188 cases in adults. Am J Respir Crit Care Med. 2003;167(5):684–9. doi: 10.1164/rccm.200206-631OC 12411286 10.1164/rccm.200206-631OC

[4] Lichtman AR, Mohrcken S, Engelbrecht M, Bigalke M. Pathophysiology of severe forms of falciparum malaria. Crit Care Med. 1990;18(6):666–8. 2188791 10.1097/00003246-199006000-00020

[5] White NJ, Ho M. The pathophysiology of malaria. Adv Parasitol. 1992;31:83–173. 1496930 10.1016/s0065-308x(08)60021-4

[6] Pongponratn E, Riganti M, Punpoowong B, Aikawa M. Microvascular sequestration of parasitized erythrocytes in human falciparum malaria: a pathological study. Am J Trop Med Hyg. 1991;44(2):168–75. 2012260 10.4269/ajtmh.1991.44.168

[7] Brooks MH, Kiel FW, Sheehy TW, Barry KG. Acute pulmonary edema in falciparum malaria. The New England Journal of Medicine. 1968;279:732–7.

[8] Feldman RM, Singer C. Noncardiogenic pulmonary edema and pulmonary fibrosis in falciparum malaria. Rev Infect Dis. 1987;9(1):134–9. 3547568 10.1093/clinids/9.1.134

[9] Tatke M, Malik GB. Pulmonary pathology in severe malaria infection in health and protein deprivation. J Trop Med Hyg. 1990;93(6):377–82. 2270000

[10] Angchaisuksiri P. Coagulopathy in malaria. Thromb Res. 2014;133(1):5–9. doi: 10.1016/j.thromres.2013.09.030 24099998 10.1016/j.thromres.2013.09.030

[11] Finigan JH. The coagulation system and pulmonary endothelial function in acute lung injury. Microvasc Res. 2009;77(1):35–8. doi: 10.1016/j.mvr.2008.09.002 18938186 10.1016/j.mvr.2008.09.002

[12] Rasmussen BS, Maltesen RG, Pedersen S, Kristensen SR. Early coagulation activation precedes the development of acute lung injury after cardiac surgery. Thromb Res. 2016;139:82–4. doi: 10.1016/j.thromres.2016.01.015 26916300 10.1016/j.thromres.2016.01.015

[13] Neyrinck AP, Liu KD, Howard JP, Matthay MA. Protective mechanisms of activated protein C in severe inflammatory disorders. Br J Pharmacol. 2009;158(4):1034–47. doi: 10.1111/j.1476-5381.2009.00251.x 19466992 10.1111/j.1476-5381.2009.00251.x PMC2785525

[14] Moxon CA, Wassmer SC, Milner DA Jr., Chisala NV, Taylor TE, Seydel KB, et al Loss of endothelial protein C receptors links coagulation and inflammation to parasite sequestration in cerebral malaria in African children. Blood. 2013;122(5):842–51. doi: 10.1182/blood-2013-03-490219 23741007 10.1182/blood-2013-03-490219 PMC3731936

[15] Aird WC, Mosnier LO, Fairhurst RM. Plasmodium falciparum picks (on) EPCR. Blood. 2014;123(2):163–7. doi: 10.1182/blood-2013-09-521005 24246501 10.1182/blood-2013-09-521005 PMC3888284

[16] Turner L, Lavstsen T, Berger SS, Wang CW, Petersen JE, Avril M, et al Severe malaria is associated with parasite binding to endothelial protein C receptor. Nature. 2013;498(7455):502–5. doi: 10.1038/nature12216 23739325 10.1038/nature12216 PMC3870021

[17] van der Poll T. The endothelial protein C receptor and malaria. Blood. 2013;122(5):624–5. doi: 10.1182/blood-2013-06-508531 23908442 10.1182/blood-2013-06-508531

[18] Naka I, Patarapotikul J, Hananantachai H, Imai H, Ohashi J. Association of the endothelial protein C receptor (PROCR) rs867186-G allele with protection from severe malaria. Malar J. 2014;13(1):105 doi: 10.1186/1475-2875-13-105 24635948 10.1186/1475-2875-13-105 PMC4004250

[19] Pongponratn E, Turner GD, Day NP, Phu NH, Simpson JA, Stepniewska K, et al An ultrastructural study of the brain in fatal Plasmodium falciparum malaria. Am J Trop Med Hyg. 2003;69(4):345–59. 14640492

[20] Fanelli V, Ranieri VM. Mechanisms and clinical consequences of acute lung injury. Ann Am Thorac Soc. 2015;12 Suppl 1:S3–8. doi: 10.1513/AnnalsATS.201407-340MG 25830831 10.1513/AnnalsATS.201407-340MG

[21] van den Brand JM, Haagmans BL, van Riel D, Osterhaus AD, Kuiken T. The pathology and pathogenesis of experimental severe acute respiratory syndrome and influenza in animal models. J Comp Pathol. 2014;151(1):83–112. doi: 10.1016/j.jcpa.2014.01.004 24581932 10.1016/j.jcpa.2014.01.004 PMC7094469

[22] Zapol WM, Trelstad RL, Coffey JW, Tsai I, Salvador RA. Pulmonary fibrosis in severe acute respiratory failure. Am Rev Respir Dis. 1979;119(4):547–54. doi: 10.1164/arrd.1979.119.4.547 443627 10.1164/arrd.1979.119.4.547

[23] Ampawong S, Chaisri U, Viriyavejakul P, Prapansilp P, Grau GE, Turner GD, et al A potential role for interleukin-33 and gamma-epithelium sodium channel in the pathogenesis of human malaria associated lung injury. Malar J. 2015;14:389 doi: 10.1186/s12936-015-0922-x 26437894 10.1186/s12936-015-0922-x PMC4595310

[24] Ayimba E, Hegewald J, Segbena AY, Gantin RG, Lechner CJ, Agosssou A, et al Proinflammatory and regulatory cytokines and chemokines in infants with uncomplicated and severe Plasmodium falciparum malaria. Clin Exp Immunol. 2011;166(2):218–26. doi: 10.1111/j.1365-2249.2011.04474.x 21985368 10.1111/j.1365-2249.2011.04474.x PMC3219897

[25] WHO. Guidelines for the treatment of malaria Switzerland: World Health Organization; 2010. 25473692

[26] Jaramillo M, Bellemare MJ, Martel C, Shio MT, Contreras AP, Godbout M, et al Synthetic Plasmodium-like hemozoin activates the immune response: a morphology—function study. PLoS One. 2009;4(9):e6957 doi: 10.1371/journal.pone.0006957 19742308 10.1371/journal.pone.0006957 PMC2734055

[27] Liu K, Liu PC, Liu R, Wu X. Dual AO/EB staining to detect apoptosis in osteosarcoma cells compared with flow cytometry. Med Sci Monit Basic Res. 2015;21:15–20. doi: 10.12659/MSMBR.893327 25664686 10.12659/MSMBR.893327 PMC4332266

[28] Srinivas R, Agarwal R, Gupta D. Severe sepsis due to severe falciparum malaria and leptospirosis co-infection treated with activated protein C. Malar J. 2007;6:42 doi: 10.1186/1475-2875-6-42 17428347 10.1186/1475-2875-6-42 PMC1950478

[29] Gillrie MR, Lee K, Gowda DC, Davis SP, Monestier M, Cui L, et al Plasmodium falciparum histones induce endothelial proinflammatory response and barrier dysfunction. Am J Pathol. 2012;180(3):1028–39. doi: 10.1016/j.ajpath.2011.11.037 22260922 10.1016/j.ajpath.2011.11.037 PMC3448071

[30] Avril M, Bernabeu M, Benjamin M, Brazier AJ, Smith JD. Interaction between Endothelial Protein C Receptor and Intercellular Adhesion Molecule 1 to Mediate Binding of Plasmodium falciparum-Infected Erythrocytes to Endothelial Cells. MBio. 2016;7(4). doi: 10.1128/mBio.00615-16 27406562 10.1128/mBio.00615-16 PMC4958245

[31] Gillrie MR, Avril M, Brazier AJ, Davis SP, Stins MF, Smith JD, et al Diverse functional outcomes of Plasmodium falciparum ligation of EPCR: potential implications for malarial pathogenesis. Cell Microbiol. 2015;17(12):1883–99. doi: 10.1111/cmi.12479 26119044 10.1111/cmi.12479 PMC4661070

[32] Mosnier LO, Lavstsen T. The role of EPCR in the pathogenesis of severe malaria. Thromb Res. 2016;141 Suppl 2:S46–9. doi: 10.1016/S0049-3848(16)30364-4 27207424 10.1016/S0049-3848(16)30364-4 PMC5481197

[33] Frank PG, Lisanti MP. ICAM-1: role in inflammation and in the regulation of vascular permeability. Am J Physiol Heart Circ Physiol. 2008;295(3):H926–H7. doi: 10.1152/ajpheart.00779.2008 18689494 10.1152/ajpheart.00779.2008 PMC2544488

[34] Greineder CF, Chacko AM, Zaytsev S, Zern BJ, Carnemolla R, Hood ED, et al Vascular immunotargeting to endothelial determinant ICAM-1 enables optimal partnering of recombinant scFv-thrombomodulin fusion with endogenous cofactor. PLoS One. 2013;8(11):e80110 doi: 10.1371/journal.pone.0080110 24244621 10.1371/journal.pone.0080110 PMC3828233

[35] Fabbi M, Carbotti G, Ferrini S. Dual Roles of IL-27 in Cancer Biology and Immunotherapy. Mediators Inflamm. 2017;2017:3958069 doi: 10.1155/2017/3958069 28255204 10.1155/2017/3958069 PMC5309407

[36] Liu L, Meng J, Zhang C, Duan Y, Zhao L, Wang S, et al Effects on apoptosis and cell cycle arrest contribute to the antitumor responses of interleukin-27 mediated by retrovirus in human pancreatic carcinoma cells. Oncol Rep. 2012;27(5):1497–503. doi: 10.3892/or.2012.1663 22293948 10.3892/or.2012.1663

[37] Percario S, Moreira DR, Gomes BA, Ferreira ME, Goncalves AC, Laurindo PS, et al Oxidative stress in malaria. Int J Mol Sci. 2012;13(12):16346–72. doi: 10.3390/ijms131216346 23208374 10.3390/ijms131216346 PMC3546694

[38] Chow CW, Herrera Abreu MT, Suzuki T, Downey GP. Oxidative stress and acute lung injury. Am J Respir Cell Mol Biol. 2003;29(4):427–31. doi: 10.1165/rcmb.F278 14500253 10.1165/rcmb.F278

[39] Cross CE, Frei B, Louie S. The adult respiratory distress syndrome (ARDS) and oxidative stress: therapeutic implications. Adv Exp Med Biol. 1990;264:435–48. 2244524 10.1007/978-1-4684-5730-8_69

[40] Scaccabarozzi D, Deroost K, Lays N, Omodeo Sale F, Van den Steen PE, Taramelli D. Altered Lipid Composition of Surfactant and Lung Tissue in Murine Experimental Malaria-Associated Acute Respiratory Distress Syndrome. PLoS One. 2015;10(12):e0143195 doi: 10.1371/journal.pone.0143195 26624290 10.1371/journal.pone.0143195 PMC4666673

[41] Deroost K, Tyberghein A, Lays N, Noppen S, Schwarzer E, Vanstreels E, et al Hemozoin induces lung inflammation and correlates with malaria-associated acute respiratory distress syndrome. Am J Respir Cell Mol Biol. 2013;48(5):589–600. doi: 10.1165/rcmb.2012-0450OC 23328641 10.1165/rcmb.2012-0450OC

[42] Milner D Jr., Factor R, Whitten R, Carr RA, Kamiza S, Pinkus G, et al Pulmonary pathology in pediatric cerebral malaria. Hum Pathol. 2013;44(12):2719–26. doi: 10.1016/j.humpath.2013.07.018 24074535 10.1016/j.humpath.2013.07.018 PMC3838443

[43] N'Dilimabaka N, Taoufiq Z, Zougbede S, Bonnefoy S, Lorthiois A, Couraud PO, et al P. falciparum isolate-specific distinct patterns of induced apoptosis in pulmonary and brain endothelial cells. PLoS One. 2014;9(3):e90692 doi: 10.1371/journal.pone.0090692 24686750 10.1371/journal.pone.0090692 PMC3970966

[44] Hashimoto S, Kobayashi A, Kooguchi K, Kitamura Y, Onodera H, Nakajima H. Upregulation of two death pathways of perforin/granzyme and FasL/Fas in septic acute respiratory distress syndrome. Am J Respir Crit Care Med. 2000;161(1):237–43. doi: 10.1164/ajrccm.161.1.9810007 10619826 10.1164/ajrccm.161.1.9810007

[45] Song Y, Mao B, Qian G. [The role of apoptosis and Fas/FasL in lung tissue in patients with acute respiratory distress syndrome]. Zhonghua Jie He He Hu Xi Za Zhi. 1999;22(10):610–2. 11776551

[46] Pino P, Vouldoukis I, Kolb JP, Mahmoudi N, Desportes-Livage I, Bricaire F, et al Plasmodium falciparum—infected erythrocyte adhesion induces caspase activation and apoptosis in human endothelial cells. J Infect Dis. 2003;187(8):1283–90. doi: 10.1086/373992 12696008 10.1086/373992

[47] Punsawad C, Viriyavejakul P, Setthapramote C, Palipoch S. Enhanced expression of Fas and FasL modulates apoptosis in the lungs of severe P. falciparum malaria patients with pulmonary edema. Int J Clin Exp Pathol. 2015;8(9):10002–13. 26617708 PMC4637523

[48] Bertin J, Guo Y, Wang L, Srinivasula SM, Jacobson MD, Poyet JL, et al CARD9 is a novel caspase recruitment domain-containing protein that interacts with BCL10/CLAP and activates NF-kappa B. J Biol Chem. 2000;275(52):41082–6. doi: 10.1074/jbc.C000726200 11053425 10.1074/jbc.C000726200

[49] Punsawad C, Maneerat Y, Chaisri U, Nantavisai K, Viriyavejakul P. Nuclear factor kappa B modulates apoptosis in the brain endothelial cells and intravascular leukocytes of fatal cerebral malaria. Malar J. 2013;12:260 doi: 10.1186/1475-2875-12-260 23890318 10.1186/1475-2875-12-260 PMC3728032

[50] Viriyavejakul P, Khachonsaksumet V, Punsawad C. Liver changes in severe Plasmodium falciparum malaria: histopathology, apoptosis and nuclear factor kappa B expression. Malar J. 2014;13:106 doi: 10.1186/1475-2875-13-106 24636003 10.1186/1475-2875-13-106 PMC3995448

[51] Jiang S, Bo L, Du X, Liu J, Zeng X, He G, et al CARD9-mediated ambient PM2.5-induced pulmonary injury is associated with Th17 cell. Toxicol Lett. 2017;273:36–43. doi: 10.1016/j.toxlet.2017.03.015 28315386 10.1016/j.toxlet.2017.03.015

[52] Uematsu T, Iizasa E, Kobayashi N, Yoshida H, Hara H. Loss of CARD9-mediated innate activation attenuates severe influenza pneumonia without compromising host viral immunity. Sci Rep. 2015;5:17577 doi: 10.1038/srep17577 26627732 10.1038/srep17577 PMC4667252

[53] Foster KA, Yazdanian M, Audus KL. Microparticulate uptake mechanisms of in-vitro cell culture models of the respiratory epithelium. J Pharm Pharmacol. 2001;53(1):57–66. 11206193 10.1211/0022357011775190

[54] Wasylnka JA, Moore MM. Uptake of Aspergillus fumigatus Conidia by phagocytic and nonphagocytic cells in vitro: quantitation using strains expressing green fluorescent protein. Infect Immun. 2002;70(6):3156–63. doi: 10.1128/IAI.70.6.3156-3163.2002 12011010 10.1128/IAI.70.6.3156-3163.2002 PMC127978

[55] Yamazaki T, Takemura H, Inoue M, Ogawa M, Ando S, Sato K, et al The intracellular accumulation of phagocytic and epithelial cells and the inhibitory effect on Chlamydophila (Chlamydia) pneumoniae of telithromycin and comparator antimicrobials. J Chemother. 2008;20(4):428–30. doi: 10.1179/joc.2008.20.4.428 18676220 10.1179/joc.2008.20.4.428

[56] Parroche P, Lauw FN, Goutagny N, Latz E, Monks BG, Visintin A, et al Malaria hemozoin is immunologically inert but radically enhances innate responses by presenting malaria DNA to Toll-like receptor 9. Proc Natl Acad Sci U S A. 2007;104(6):1919–24. doi: 10.1073/pnas.0608745104 17261807 10.1073/pnas.0608745104 PMC1794278

